# Notch1-CD22-Dependent Immune Dysregulation in the SARS-CoV2-Associated Multisystem Inflammatory Syndrome in Children

**DOI:** 10.21203/rs.3.rs-1054453/v1

**Published:** 2022-04-11

**Authors:** Talal A. Chatila, Mehdi Benamar, Qian Chen, Janet Chou, Amelie Julé, Rafik Boudra, Paola Contini, Elena Crestani, Muyun Wang, Jason Fong, Peggy Lai, Shira Rockwitz, Pui Lee, Tsz Man Fion Chan, Ekin Zeynep Altun, Eda Kepenekli, Elif Karakoc-Aydiner, Ahmet Ozen, Perran Boran, Fatih Aygun, Pinar Onal, Ayse Ayzit Kilinc Sakalli, Haluk Cokugras, Metin Gelmez, Fatma Öktelik, Esin Aktaş Cetin, Yuelin Zhong, Maria Taylor, Katherine Irby, Natasha Halasa, Sara Signa, Ignazia Prigione, Marco Gattorno, Nicola Cotugno, Donato Amodio, Raif Geha, Mary Beth Son, Jane Newburger, Pankaj Agrawal, Stefano Volpi, Paolo Palma, Ayca Kiykim, Adrienne Randolph, Gunnur Deniz, Safa Baris, Raffaele De Palma, Klaus Schmitz-Abe, Louis-Marie Charbonnier, Lauren Henderson

**Affiliations:** Boston Children’s Hospital - Harvard Medical School; INSERM; Division of Immunology, Boston Children’s Hospital, Boston, Massachusetts, USA; Department of Pediatrics, Harvard Medical School, Boston, Massachusetts, USA;; Childrens Hospital Boston/Harvard Medical School; Division of Immunology, Boston Children’s Hospital, Boston, Massachusetts, USA; Department of Pediatrics, Harvard Medical School, Boston, Massachusetts, USA;; Harvard Medical School; University of Genoa; Boston Children’s Hospital - Harvard Medical School; 1Division of Immunology, Boston Children’s Hospital, Boston, Massachusetts, USA; 2Department of Pediatrics, Harvard Medical School, Boston, Massachusetts, USA;; Boston Children’s Hospital; Massachusetts General Hospital, Harvard Medical School; The Manton Center for Orphan Disease Research, Boston Children’s Hospital, Boston, USA; Boston Children’s Hospital; 1Division of Immunology, Boston Children’s Hospital, Boston, Massachusetts, USA; 2Department of Pediatrics, Harvard Medical School, Boston, Massachusetts, USA;; Ministry of Healthy, Marmara University Education and Training Hospital, Department of Pediatrics, Istanbul, Turkey; Marmara University, Faculty of Medicine, Division of Pediatric Infectious Diseases, Istanbul, Turkey; Marmara University, School of Medicine, Department of Pediatrics, Division of Allergy and Immunology, Istanbul; Marmara University; Marmara University, Faculty of Medicine, Division of Social Pediatrics, Istanbul, Turkey; Department of Pediatric Intensive Care, Cerrahpaşa Faculty of Medicine, İstanbul University-Cerrahpaşa, İstanbul, Turkey.; Department of Pediatric Infectious Diseases, Cerrahpaşa Faculty of Medicine, İstanbul University-Cerrahpaşa, İstanbul, Turkey.; Department of Pediatric Pulmonology, Cerrahpaşa Faculty of Medicine, İstanbul University-Cerrahpaşa, İstanbul, Turkey.; Department of Pediatric Infectious Diseases, Cerrahpaşa Faculty of Medicine, İstanbul University-Cerrahpaşa, İstanbul, Turkey. Department of Pediatric Pulmonology, Cerrahp; Istanbul University; Istanbul University; Istanbul University; 1Division of Immunology, Boston Children’s Hospital, Boston, Massachusetts, USA; 2Department of Pediatrics, Harvard Medical School, Boston, Massachusetts, USA;; 1Division of Immunology, Boston Children’s Hospital, Boston, Massachusetts, USA; 2Department of Pediatrics, Harvard Medical School, Boston, Massachusetts, USA;; Arkansas Children’s Hospital; Vanderbilt University Medical Center; DINOGMI, Università degli Studi di Genova, Genova, Italy and Center for Autoinflammatory Diseases and Immunodeficiencies, IRCCS Istituto Giannina Gaslini, Genova, Italy; Center for Autoinflammatory Diseases and Immunodeficiencies, IRCCS Istituto Giannina Gaslini, Genova, Italy; Center for Autoinflammatory Diseases and Immunodeficiencies, IRCCS Istituto Giannina Gaslini, Genova, Italy; Clinical and Research Unit of Clinical Immunology and Vaccinology, Bambino Gesù Children’s Hospital, IRCCS, Rome, Italy, Chair of Pediatrics, Department of Systems Medicine, University; Clinical and Research Unit of Clinical Immunology and Vaccinology, Bambino Gesù Children’s Hospital, IRCCS, Rome, Italy,; Childrens Hospital Boston/Harvard Medical School; Boston Children’s Hospital; Boston Children’s Hospital; Division of Newborn Medicine and Genetics & Genomics, Department of Pediatrics, Boston Children’s Hospital, Harvard Medical School, Boston, Massachusetts, USA; DINOGMI, Università degli Studi di Genova, Genova, Italy and Center for Autoinflammatory Diseases and Immunodeficiencies, IRCCS Istituto Giannina Gaslini, Genova, Italy; Clinical and Research Unit of Clinical Immunology and Vaccinology, Bambino Gesù Children’s Hospital, IRCCS, Rome, Italy, Chair of Pediatrics, Department of Systems Medicine, University; Division of Pediatric Allergy and Immunology, Faculty of Medicine, Istanbul University-Cerrahpasa, Istanbul, Turkey;; Boston Children’s Hospital; Istanbul University; Marmara University, School of Medicine, Department of Pediatrics, Division of Allergy and Immunology, Istanbul; University of Genova; 1Division of Immunology, Boston Children’s Hospital, Boston, Massachusetts, USA; 2Department of Pediatrics, Harvard Medical School, Boston, Massachusetts, USA;; Boston Children’s Hospital, Boston; Boston Children’s Hospital and Harvard Medical School

## Abstract

Multisystem inflammatory syndrome in children (MIS-C) evolves in some pediatric patients following acute infection with SARS-CoV-2 by hitherto unknown mechanisms. Whereas acute-COVID-19 severity and outcome were previously correlated with Notch4 expression on regulatory T (Treg) cells, here we show that the Treg cells in MIS-C are destabilized in association with increased Notch1 expression. Genetic analysis revealed that MIS-C patients were enriched in rare deleterious variant impacting inflammation and autoimmunity pathways, including dominant negative mutations in the Notch1 regulators *NUMB* and *NUMBL*. Notch1 signaling in Treg cells induced CD22, leading to their destabilization in an mTORC1 dependent manner and to the promotion of systemic inflammation. These results establish a Notch1-CD22 signaling axis that disrupts Treg cell function in MIS-C and point to distinct immune checkpoints controlled by individual Treg cell Notch receptors that shape the inflammatory outcome in SARS-CoV-2 infection.

## Introduction

COVID-19, caused by the severe acute respiratory syndrome coronavirus 2 (SARS-CoV-2), has resulted in massive morbidity and mortality worldwide^1, 2^. Acute infection is associated in some subjects with pneumonia and marked hypoxia, leading to acute respiratory distress syndrome as well as other life-threatening complications^3, 4, 5^. This inflammation critically involves a dysregulated immune response characterized by intense activation of innate and adaptive immunity associated with features of a cytokine storm^6, 7^. While most patients recover from this acute infection, a subset develops persistent symptoms related to different organ system dysfunction including the respiratory, cardiovascular, gastrointestinal, renal, and central nervous systems^8^.

A special case in point is the course of SARS-CoV-2 infection in children. While most children remain asymptomatic or develop from mild infection, some develop a multi-system inflammatory syndrome in children (MIS-C) approximately one month after initial infection^9, 10, 11, 12, 13^. These patients exhibit severe immune dysregulation characterized by intense cytokine production and lymphocyte activation associated with fever and end-organ dysfunction including mucocutaneous, cardiovascular, hematologic, and especially gastrointestinal systems^14, 15, 16, 17, 18, 19, 20, 21^. In particular, IFNg has been identified as a key cytokine in MIS-C with increased levels associated with disease severity and increased organ system involvement^22, 23^. There are defining characteristics of MIS-C that remain perplexing, including the substantial delay between the initial SARS-CoV-2 infection and MIS-C^9, 10, 12^. Unlike children with acute COVID-19 pneumonia, most patients with MIS-C are previously healthy and are able to mount a robust immune response to SARS-CoV-2 with neutralizing antibodies to the virus^11, 18, 24^. This constellation of features in MIS-C suggests that an evolving hyperinflammatory immune response to SARS-CoV-2 is part of the pathophysiology of this syndrome. Indeed, studies based on relatively small number of patients suggests that a genetic predisposition may contribute to the immune dysregulation in MIS-C^25, 26^.

Notch signaling pathways have emerged as important regulators of the immune system by influencing both Treg and Tconv cells responses^27, 28^. In mammals, the Notch family is composed by 4 Notch receptors (Notch1-4) and 5 ligands (Delta-like1, 3, and 4 and Jagged1 and 2)^29^. Recent studies have outlined a prominent role for NOTCH4 in the immune dysregulation in acute COVID19 and related respiratory viral illnesses^30^. Notch4 is upregulated on lung tissue Treg cells in an IL-6-dependent manner to subvert their tissue repair function in favor of an inflammatory response^30, 31, 32^. The *NOTCH4* locus is associated with critical illness in COVID-19^33^. However, the immune dysregulatory mechanisms operative in post-acute COVID19 syndromes including MIS-C remain unclear.

In this study, we demonstrate that while NOTCH4 is also upregulated on circulating Treg cells of children with acute COVID19 as a function of disease severity, the Treg cells in MIS-C additionally upregulate NOTCH1 expression, a pathway previously implicated in Th1 type immune dysregulation, autoimmunity, graft versus host disease and solid organ rejection^34, 35^. Gene enrichment using whole genome/exome sequence analysis employing Fischer testing and Monte-Carlo simulation revealed the enrichment in MIS-C patients of rare mutations impacting pathways of inflammation and autoimmunity, many of which contained Notch-related genes. Consistent with these results, a loss of function mutation was identified in the negative NOTCH1 regulator *NUMB*^36^. In mice expressing an active form of Notch1 in Treg cells (*Foxp3*^EGFPCre^*R26*^N1c^), treatment with Poly I:C to simulate viral infection induced systemic inflammation that recapitulated the phenotype of MIS-C. Notch1 signaling in Treg cells induced the B cell inhibitory receptor CD22^37, 38^, which promoted systemic inflammation in association with the expression of the α4β7 gut homing receptor. CD22 destabilized Treg cells and impaired their suppressive function in an mTORC1-dependent manner. Treatment of mice with an anti-CD22 mAb suppressed the development of systemic inflammation following Poly I:C treatment by restoring the Treg cells suppressive function. These findings point to the mobilization of Treg cell-specific tissue inflammatory licensing modules involving different Notch receptors that is operative in MIS-C and point to interventions along the Notch1-CD22 axis as therapeutic strategy in MIS-C.

## Results

### Increased CD4 ^+^ T cell activation and Treg cell destabilization in MIS-C.

To elucidate the immune dysregulatory mechanisms operative in MIS-C, we studied an international cohort of 45 children with MIS-C and 50 children with COVID-19 from centers in the United States, Italy and Turkey (Table 1 and Patient Cohorts section in Methods). For comparison, 5 children with Kawasaki disease (KD), 12 adults with COVID-19, and 18 pediatric healthy controls were also evaluated. All MIS-C patients met the Centers for Disease Control (CDC) Case Definition for MIS-C while 93% fulfilled the WHO case definition^39, 40^. Fever was universal in MIS-C patients and rash (49%), conjunctivitis (58%), and GI symptoms (96%) were also common. Children with MIS-C were highly inflamed (median CRP 16.0 mg/dL, IQR 7.8–24.0), lymphopenic (median absolute lymphocyte count 0.91 ×10^3^/mL, IQR 0.53–1.35) and coagulopathic (median D-dimer 3.1 mcg/mL, IQR 1.5–6.2). Over 90% of MI S-C patients demonstrated positive SARS-CoV-2 serologies. 18/45 (40%) were considered to have severe MIS-C defined by admission to the intensive care unit (ICU), need for vasopressor support, and/or development of coronary artery aneurysms. The demographics and key clinical findings in the respective patient groups are delineated in **Supplementary Table 1**.

**Figure 1:**
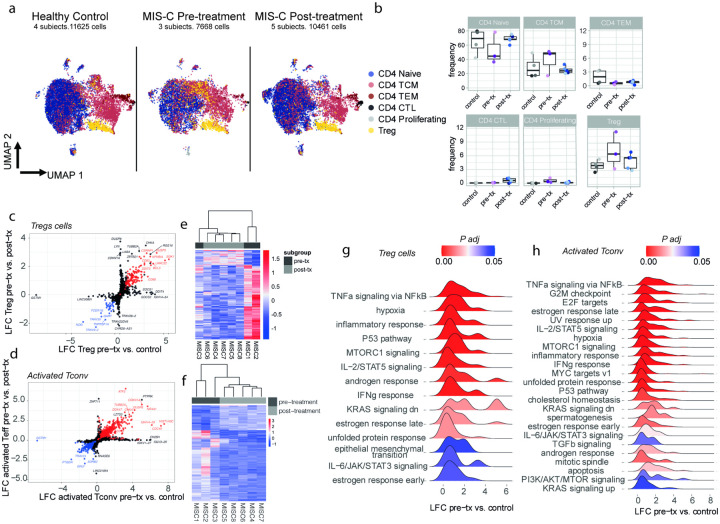
Increased Notch1 expression on circulating CD4^+^ Treg and Tconv cells in MIS-C. **a-f**, Flow cytometric analysis, cell frequencies and mean fluorescence intensity (MFI) of Notch1 (**a-c**) and Notch4 expression (**d-f**) in CD4^+^ Treg and Tconv cells of healthy control subjects, and patients with Kawasaki disease, adult subjects with severe COVID-19, pediatric subjects with mild or severe COVID-19 and MIS-C subjects (healthy controls n=18, Kawasaki disease n=5, severe adult COVID-19 n=12, mild pediatric COVID-19 n=21, severe pediatric COVID-19 n=4, and MIS-C n=29). **g**. Co-expression of Notch1 and Notch2 and Notch1 and Notch4 on circulating Treg cells of MIS-C subjects. **h**, serum concentrations of IL-1b, IL-6, TNF, IFNa, IFNl2/3, IFNg IL-10 and IP-10 in control and the respective patient group subjects (healthy controls n=10, mild pediatric COVID-19 n=25, severe pediatric COVID-19 n=4, and MIS-C n=25). **i**. Flow cytometric analysis and frequencies of Notch1 expression on ant-CD3-anti-CD28-activated CD4^+^ human Treg cells treated with the respectively indicated cytokines. Each symbol represents one subject. Numbers in flow plots indicate percentages. Error bars indicate SEM. Statistical tests: *P<0.05, **P<0.01, ****P<0.0001 by one-way ANOVA with Dunnett’s post hoc analysis (**a-e; g**). Data representative of two or three independent experiments.

To further delineate the CD4^+^ T cell dynamics in MIS-C, we carried out single-cell RNA sequencing (scRNA-seq) analysis on CD4^+^ T cells from the peripheral blood of four healthy controls, three MIS-C patients sampled prior to treatment and another five MIS-C patients sampled post-treatment. To characterize the sub-populations of CD4^+^ T cells in this high-dimensional analysis, we first mapped our transcriptomic data to a reference human PBMC dataset using Azimuth^41^, thereby delineating 6 subsets of CD4^+^ T cells (Fig. 1a,b). To further elucidate the heterogeneity in our dataset, we performed a graph-based clustering analysis using Seurat, which uncovered 16 clusters. Eight of these clusters (Clusters 1 to 8) were enriched in cells annotated as CD4 naïve by Azimuth and expressing genes associated with a naïve CD4^+^ T cell profile (e.g., *CCR7* and *SELL*), 5 (Clusters 10 to 14) were enriched in activated CD4^+^ T cells (*CD69*), including one with high NF-kB signaling (Cluster 10; *NFKB1*). The final 3 clusters encompassed a mix of naïve and activated cells, including one cluster delineated by viral sensing gene transcripts (Cluster 9; *IFIT2*, *IFIT3*), one cluster enriched in Treg cell transcripts (Cluster 15; *FOXP3*) and another with mitotic cells (Cluster 16; *TRBC1*) (**Extended Data** Fig. 1a-f). Prior to treatment, MIS-C patients exhibited prominent expansion of cluster 10, enclosing both cells annotated as Tconv and Tregs by Azimuth. Cluster 10 was characterized by increased *NFKB1* expression and NF-κB signaling and contracted following immunomodulatory therapy (**Extended Data** Fig. 1a-f). To further decipher differences in CD4^+^ T cell transcriptomic programs between patient groups, we also performed pseudobulk differential analysis (DEA) with a focus on both Treg (cells found in Cluster 15 or delineated as Tregs by Azimuth) and Tconv cells (cells found in clusters 9 to 14 and delineated as activated Tconv by Azimuth). For this pseudobulk DEA, we aggregated gene expression data at the patient level for Tregs and activated Tconv, and performed pairwise comparisons of MIS-C pre-treatment, post-treatment and control groups using DESeq2. The DEA were followed by gene set enrichment analyses (GSEA) against the MSigDB Hallmark collection and using the ranked log2 fold changes as input, which reinforced our prior observations of NF-kB pathway activation in pre-treatment MIS-C samples, not only in Tconv but also in Tregs (Fig. 1c–h). Moreover, we also found new pathways that were up regulated in the MIS-C pre-treatment group, including for example MTORC1 and P53 pathway (Fig. 1c–h). These results indicated that MIS-C is associated with enhanced Tconv activation and Treg dysregulation.

### Increased NOTCH1 expression on CD4 ^+^ Treg and Tconv cells in MIS-C.

**Figure 2:**
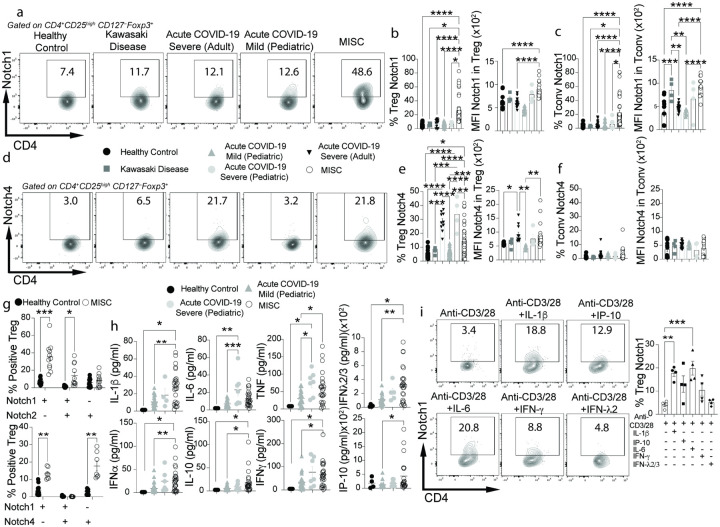
Increased Notch1 expression on circulating CD4^+^ Treg and Tconv cells in MIS-C. **a-f**, Flow cytometric analysis, cell frequencies and mean fluorescence intensity (MFI) of Notch1 (**a-c**) and Notch4 expression (**d-f**) in CD4^+^ Treg and Tconv cells of healthy control subjects, and patients with Kawasaki disease, adult subjects with severe COVID-19, pediatric subjects with mild or severe COVID-19 and MIS-C subjects (healthy controls n=18, Kawasaki disease n=5, severe adult COVID-19 n=12, mild pediatric COVID-19 n=21, severe pediatric COVID-19 n=4, and MIS-C n=29). **g**. Co-expression of Notch1 and Notch2 and Notch1 and Notch4 on circulating Treg cells of MIS-C subjects. **h**, serum concentrations of IL-1b, IL-6, TNF, IFNa, IFNl2/3, IFNg IL-10 and IP-10 in control and the respective patient group subjects (healthy controls n=10, mild pediatric COVID-19 n=25, severe pediatric COVID-19 n=4, and MIS-C n=25). **i**. Flow cytometric analysis and frequencies of Notch1 expression on ant-CD3-anti-CD28-activated CD4^+^ human Treg cells treated with the respectively indicated cytokines. Each symbol represents one subject. Numbers in flow plots indicate percentages. Error bars indicate SEM. Statistical tests: *P<0.05, **P<0.01, ****P<0.0001 by one-way ANOVA with Dunnett’s post hoc analysis (**a-e; g**). Data representative of two or three independent experiments.

Previous studies have demonstrated a key role for Notch signaling-mediated Teg cell dysregulation in licensing tissue inflammation^30, 32, 34, 35^. A case in point is the upregulation of Notch4 on lung tissue Treg cells in viral infections mediated by SARS-CoV2 and influenza, leading to enahcned tissue inflammation and disease severity^30^. We analyzed the expression of different Notch receptors on CD4^+^ Treg and Tconv cells in pediatric subjects with mild and severe COVID19 and those with MIS-C. As comparison groups we included healthy children, adults with severe COVID19 and children with KD, some of whose clinical features overlap with those of MIS-C^16, 25, 39, 40^. There was marked increase in Notch1 expression on both Treg and Tconv cells of patients with MIS-C but not on those of other subject groups (Fig. 2a–c). Our previous studies have identified Notch4^+^ Treg cells to emerge in the context of lung inflammation in COVID-19 subjects and related mouse models^30^. Notch4 expression was also selectively increased on the circulating Treg cells of adult and pediatric subjects with severe COVID-19 and with MIS-C but not on their Tconv cells. Notch4 was also not upregulated on Treg cells of patients with mild COVID19 or with KD (Fig. 2d–f). Expression of Notch1 and Notch4 on Treg cells of MIS-C patients was non-overlapping, suggesting that they may represent distinct Treg cell populations possibly arising in different tissues (Fig. 2g, **lower panel**). In contrast, Notch2 expression was increased on Notch1^+^ Treg and Tconv cells of MIS-C subjects albeit at a lower magnitude than that of Notch1, while there was no difference in the Notch2 single positive Treg and Tconv cell populations between MIS-C and healthy controls (Fig. 2g **higher panel and Extended Data** Fig. 2a,b). Also, there was no difference in Notch3 expression between the circulating Treg and Tconv cells of different patient populations and control subjects (**Extended Data** Fig. 2c,d). Overall, these results identified increased Notch1 expression on Treg and Tconv cells as a distinguishing feature of pediatric patients with MIS-C.

Analysis of T cell phenotype revealed that MIS-C patients present a decrease of naive Tconv and Treg cells associated with an increase of activated T cells (**Extended Data** Fig. 2e-h). Analysis of the serum cytokine profile of the different patient groups revealed increased expression of IP-10, IL-1β, IL-6 and IFNλ2/3 in MIS-C and to a lesser extent in severe COVID-19 compared to controls (Fig. 2h, **Extended Data** Fig. 2i). Further analysis showed that Treg and Tconv cells of patients with MIS-C versus severe COVID-19, KD and control subjects had increased IFN-γ production (**Extended Data** Fig. 2j,k). Notably, IFN-γ expression was selectively increased in Treg cells of MIS-C subjects, while IFN-γ expression in Tconv cells was common to both severe COVID-19 and MIS-C (**Extended Data** Fig. 2j,k).. We analyzed the capacity of different cytokines found increased in the sera of MIS-C subjects to induce Notch1 expression on cell-sorted CD4^+^CD25^+^CD127^−^ human Treg cells from control subjects. Results showed that IL-1β and IL-6, and to a lesser extent IFN-γ and IP-10 all induced increased Notch1 expression on human Treg cells (Fig. 2i). Overall, these results linked the upregulation of Notch1 expression on CD4^+^ Treg and Tconv cells with the development of MIS-C.

### Identification of Notch pathway genetic variants in MIS-C.

**Figure 3:**
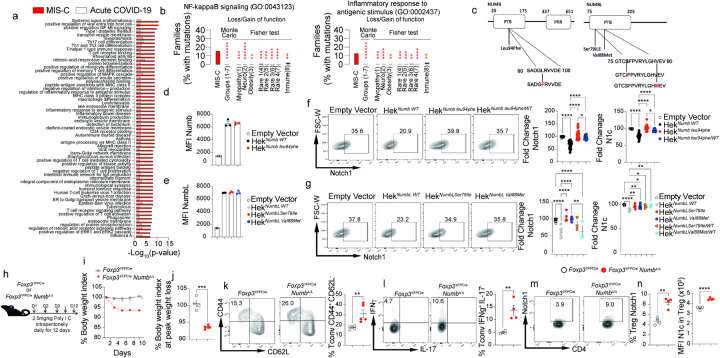
Identification of Notch pathway genetic variants in MIS-C. **a,b**. KEGG and GO pathways differentially enriched in rare mutations in MIS-C versus Acute COVID-19 by Monte Carlo simulation and Fished Test.as described in the Material’s and Methods section. **b**. Frequency of mutations in two representative pathways (“positive regulation of NF-kB signaling” and “inflammatory response to antigenic stimulus”) identified in A versus other disease groups either collectively by Monte Carlo simulation or individually by Fischer test. **c**. Schematic representation of *NUMB* and *NUMBL* mutations identified in MIS-C subjects. **d,e**. Expression of recombinant wild type NUMB protein (NUMB^WT^) and NUMB^Leu94Phe^ (D), and NUMBL^WT^, NUMBL^Ser79Ile^ and NUMBL^Val88Met^ proteins (E) in NUMB/NUMBL-deficient HEK293 cells. **f,g**. Flow cytometric analysis and fold expression of NOTCH1 and N1c in NUMB/NUMBL-deficient HEK293 cell transfected with either NUMB^Leu94Phe^ (F), NUMBL^Ser79Ile^ or NUMBL^Val88Met^ proteins (G) either alone or together with the respective WT proteins. **h**. Scheme of mouse Poly IC treatment. **i,j**. Body weight index change (I) and peak weight loss (J) of the *Foxp3*^YFPCre^ and *Foxp3*^YFPCre^*Numb*^Δ/Δ^ mice treated with Poly IC. **k,l**. Flow cytometric analysis and cell frequencies of CD44^+^CD62L^–^ (**k**) and IFNg^+^IL-17^–^ Tconv cells (**l**). **m, n**. Flow cytometric analysis (**m**) and frequencies of Notch1^+^ and Notch1c^+^ Treg cells Error bars indicate SEM. Statistical tests for panels **d-f**: *P<0.05, **P<0.01, ***P<0.001, ****P<0.0001 by one-way ANOVA with Dunnett’s post hoc analysis.

To investigate underlying genetic factors that may predispose to MIS-C versus acute pediatric COVID-19, we performed gene-enrichment tests for rare variants (stop-gain/start-loss, frameshift deletions/insertions and canonical splicing mutations) using 8626 pathways from Gene-Ontology (GO) and Kyoto Encyclopedia of Genes and Genomes (KEGG) databases (8,299 and 327 respectively). We collected genome and exome sequences on 39 MIS-C and 24 acute pediatric COVID-19 subjects, which we compared with 8 different datasets comprising 4,682 exomes collected at the Boston Children’s Hospital, including 4 rare disease categories, obesity, myopathy, autism-ADHD and Immune deficiency/dysregulation^42^(see Methods section). All samples were processed using the Variant Explorer (VExP) Pipeline with the same set parameters to avoid bias in the selection of the rare variants^43^. We performed a Fisher test for each group in the different GO and KEGG pathways. Furthermore, we validated these results by Monte-Carlo simulation testing as an unbiased stochastic approach to test for enrichment in genetic variants along individual pathways in the MIS-C group versus the sum total of the clinical comparison groups used for the Fischer tests, as described in the Methods section. Results showed that several inflammation and autoimmunity pathways were significantly enriched in rare mutations (≤ 10 in 280,000 chromosomes) in MIS-C versus acute pediatric COVID-19 and the other comparison groups (Fig. 3a,b, **Dataset 1**). Several of those pathways contained NOTCH related genes. Specific NOTCH pathway mutations predicted to be damaging and linked to those pathways were identified included *NOTCH2*, *NOTCH4* and *RPBJL* (**Dataset 2**). Overall, these results supported the presence of an underlying genetic predisposition for MIS-C.

To validate the above findings from our initial cohort, we screened 88 additional patients with MIS-C from the U.S. multicenter Overcoming COVID-19 Network for mutations in Notch-related genes (see methods in Supplement)^9, 44^. Rare damaging mutations were identified in *NUMB* and *NUMBL*, encoding closely conserved eponymous proteins that negatively regulate Notch receptor signaling and trafficking^36, 45, 46, 47^. We further analyzed three mutation in the phosphotyrosine binding (PTB) domain of NUMB (*NM_001005745.1:c.280C > T; p.Leu94Phe), NUMBL (NM_004756.5:c.236G > T,NP_004747.1:p.Ser79Ile) and NUMBL (NM_004756.5:c.262G > A,NP_004747.1:p.Val88Met*) (Fig. 3c **and Dataset 3**). These mutations, which were either not found in the Genome Aggregation Database (gnomAD) (NUMB^Leu94Phe^ and NUMBL^Ser79IIe^; gnomAD = 0) or very rarely so (NUMBL^Val88Met^; gnomAD = 4), were predicted to impair NUMB and NUMBL regulatory functions^36, 46, 47^. This prediction was tested by analyzing the impact of the respective NUMB/NUMBL mutations on NOTCH1 expression and function. Transgenic expression of the respective mutant protein in NUMB/NUMBL-deficient human embryonic kidney 293 (HEK293) cells revealed that their expression was similar to that of wild-type NUMB (NUMB^WT^) or NUMBL (NUMBL^WT^) protein (Fig. 3d,e). However, whereas transgenic NUMB^WT^ or NUMBL^WT^ decreased the expression of NOTCH1 in HEK293 cells by 32%, the NUMB^Leu94Phe^, NUMBL^Ser79IIe^ and NUMBL^Val88Met^ mutant completely failed to do so. Similarly, the NUMB^Leu94Phe^, NUMBL^Ser79IIe^ and NUMBL^Val88Met^ mutant failed to decrease nuclear NOTCH1 cytoplasmic domain (N1c) localization compared to NUMB^WT^ (Fig. 3f, g). Moreover, Co-transfection of NUMB^WT^- NUMB^Leu94Phe^ and NUMBL^WT^- NUMBL^Ser79IIe^ revealed that these mutations behaved as dominant negative mutations (Fig. 3f, g). These results established that the identified NUMB/NUMBL mutations were functionally deleterious.

To further delineate the mechanisms by which deficient NUMB activity in Treg cells promotes MIS-C, we employed a mouse model in which a floxed *Numb* allele was conditionally deleted in Treg cells using a *Foxp3* promoter regulated Cre recombinase fused with YFP (*Foxp3*^YFPCre^*NUMB*^D/D^) is) (Fig. 3h). Treatment of *Foxp3*^YFPCre^*NUMB*^D/D^ with polyinosinic:polycytidylic acid (poly(I:C)), a proxy model of infection with RNA viruses^30, 48, 49, 50^, resulted in progressive weight loss. In contrast, Poly I:C-treated control *Foxp3*^YFPCre^ were minimally affected (Fig. 3i,j). Analysis of CD4^+^ T cells of *Foxp3*^YFPCre^NUMB^D/D^ mice revealed that their activation phenotype recapitulated that of CD4^+^ T cells of MIS-C patients, including increased memory markers (CD44^+^CD62L^−^) and heightened IFNγ production by both Treg and Tconv cells (Fig. 3k,l). Finally, deficiency of NUMB in Treg cell also recapitulate the upregulation of Notch1 and N1c found in MIS-C patients (Fig. 3m,n). Overall, these results indicated that MIS-C subjects may harbor mutations in the Notch pathway that contribute to disease pathogenesis.

### Poly I:C-induced multiorgan inflammatory disease in Foxp3^EGFPCre^R26^N1c/+^ mice.

**Figure 4:**
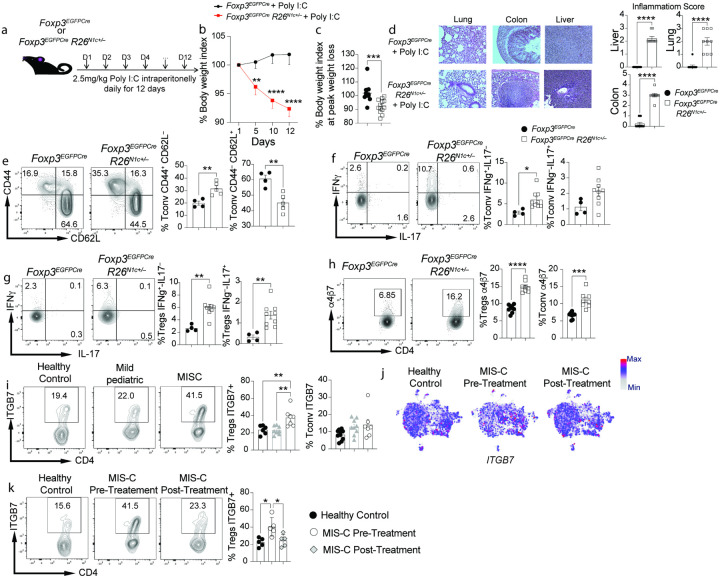
Poly I:C-induced multiorgan inflammatory disease in *Foxp3*^EGFPCre^*R26*^N1c/+^ mice. **a**. Experimental scheme. Mice were injected with Poly I:C intraperitoneally (i.p.) daily for 12 days. **b**, Weight indices of *Foxp3*^EGFPCre^ and *Foxp3*^EGFPCre^*R26*^N1c/+^ mice subjected to Poly I:C treatment. **c, d**, Hematoxylin and eosin-stained sections and inflammation score of liver, gut and lung tissues isolated from the indicated mouse groups (×200 magnification). **e**. Flow cytometric analysis and graphical representation of naïve (CD4^+^CD44^–^CD62L^+^) and activated (CD4^+^CD44^+^CD62L^–^) Tconv cells. **f,g**. Flow cytometric analysis and graphical representation of IFNg and IL-17 expression in Tconv (**f**) and Treg cells (**g**) in the respective poly I:C-treated mouse groups. **h**. Flow cytometric analysis and graphical representation of a4b7 expression in Treg and Tconv cells of the indicated mouse groups. **i**. Flow cytometric analysis and graphical representation of a4b7 expression in Treg and Tconv cells of the indicated subject groups. **j**. Relative expression of *ITGB7* in the different clusters inferred from scRNA-seq data. **k**. Flow cytometric analysis and cell frequencies of a4b7 expression on circulating CD4^+^FOXP3^+^ Treg cells in healthy control subjects and in MIS-C patients pre and post-treatment. Each symbol represents one mouse (**b-i**), one cell (**j**) or one human subject (**i,k**). Numbers in flow plots indicate percentages. Error bars indicate SEM. Statistical tests: Two-way ANOVA with Sidak’s post hoc analysis (**b**)**; Student T-test (c,d)** One-way ANOVA with Dunnett’s post hoc analysis (**e-i,k**). *P<0.05, **P<0.01, ***P<0.001, ****P<0.0001.

To further delineate the mechanisms by which increased Notch1 signaling in CD4^+^ T cells promotes MIS-C, and in view of the critical role played by Treg cells in licensing Notch1-dependent immune dysregulation^34, 35^, we employed a mouse model in which the intracellular domain of Notch1 is conditionally expressed from the Rosa26 locus (*R26*^N1c/+^) in Treg cells using a *Foxp3* promoter-regulated Cre recombinase fused with EGFP (*Foxp3*^EGFPCre^) (Fig. 4a)^34^. Treatment of *Foxp3*^EGFPCre^*R26*^N1c/+^ mice with Poly I:C intraperitoneally resulted in progressive weight loss and multi-organ inflammation. In contrast, Poly I:C-treated control *Foxp3*^EGFPCre^ were minimally affected (Fig. 4b–d). Analysis of CD4^+^ T cells of *Foxp3*^EGFPCre^*R26*^N1c/+^ mice revealed that their activation phenotype recapitulated that of CD4^+^ T cells of MIS-C patients, including increased memory markers (CD44^+^CD62L^−^) and heightened IFNγ production by both Treg and Tconv cells (Fig. 4e–g).

Most MIS-C patients present with gastrointestinal symptoms (Table 1)^14 13^. Notably, the Treg and to a lesser extent the Tconv cells of the *Foxp3*^EGFPCre^*R26*^N1c/+^ mice had increased expression of the gut homing integrin α4β7 (Fig. 4h). Increased expression of integrin α4β7 was also observed on the circulating Treg cells of MIS-C but not acute pediatric COVID-19 subjects, in agreement with a critical role of Notch1 in driving the expression of this marker (Fig. 4i). Consistent with this finding, MIS-C patients exhibited an increase in CD62L^−^CD38^+^ mucosally imprinted Treg cells (**Extended Data** Fig. 3a)^51, 52^. sc-RNA seq analysis demonstrated increased *ITGB7* transcripts in the clusters that also present an increase in Notch1 (Fig. 4j **and Extended Data** Fig. 3b). Expression of integrin α4β7 on Treg cells of MIS-C subjects declined post-treatment, in synchrony with decreased Notch1 expression (Fig. 4k). These results indicated that increased Notch1 activity in Treg cells predisposes to multi organ inflammation in the context of a viral trigger and promotes Treg cell gut homing.

### Notch1-mediated CD22 upregulation on Treg cells promotes multi organ inflammation.

**Figure 5:**
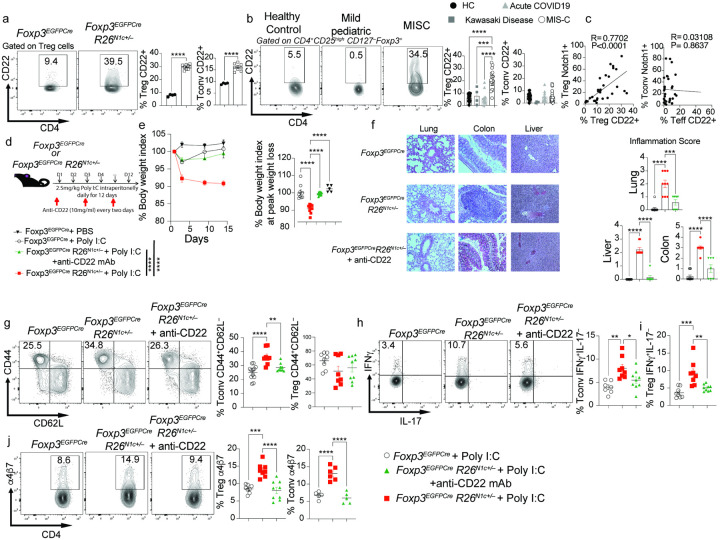
A Treg cell Notch1-CD22 axis promotes multi organ inflammation. **a**. Flow cytometric analysis and graphical representation of CD22 expression in splenic Treg and Tconv cells of *Foxp3*^EGFPCre^ and *Foxp3*^EGFPCre^*R26*^N1c/+^ mice. **b**. Flow cytometric analysis and cell frequencies of CD22 expression on circulating CD4^+^FOXP3^+^ Treg and CD4^+^FOXP3^–^ Tconv cells in healthy controls and in subjects with either mild pediatric COVID or MIS-C. **c**. Correlation analysis of CD22 expression on Treg and Tconv cells of MIS-C and control subjects as a function of Notch1 expression on these cells (n=32). **d**. Experimental scheme. Mice were injected with Poly I:C intraperitoneally (i.p.) daily for 12 days. **e**, Weight indices of *Foxp3*^EGFPCre^ and *Foxp3*^EGFPCre^*R26*^N1c/+^ mice subjected to Poly I:C treatment. **f**, Hematoxylin and eosin-stained sections and inflammation score of liver, gut and lung tissues isolated from the indicated mouse groups (×200 magnification). **g**, Flow cytometric analysis and graphical representation of naïve (CD4^+^CD44^–^ CD62L^+^) and activated (CD4^+^CD44^+^CD62L^–^) Tconv cells. **h,i** Flow cytometric analysis and graphical representation of IFNg and IL-17 expression in Tconv (H) and Treg cells (I) in the respective poly I:C-treated mouse groups. **j**. Flow cytometric analysis and graphical representation of a4b7 expression in Treg and Tconv cells of the indicated mouse groups. Each symbol represents one mouse (a, d, e-h), or one human subject (b, c). Numbers in flow plots indicate percentages. Error bars indicate SEM. Statistical tests: Student t-test (**a**);Two-way ANOVA with Sidak’s post hoc analysis (**b**); One-way ANOVA with Dunnett’s post hoc analysis (**e,g,h,i,j**); and Pearson correlation analysis (**b**). *P<0.05, **P<0.01, ***P<0.001, ****P<0.0001.

To delineate the mechanisms by which Notch1 signaling in Treg cells promotes multi organ inflammation in the context of a viral trigger, we analyzed the transcriptome of Notch1c-expressing Treg cells for pathways involved in the immune dysregulation^34^. We found upregulation of CD22, a member of the Siglec family of lectins normally found in B cells, where it acts to regulate B cell receptor signaling^38^. In particular, CD22 directs B cells to the intestinal lymphoid and mucosal tissues by upregulating the expression of the gut homing receptor α4β7^37^. Flow cytometric analysis of Treg cells of *Foxp3*^EGFPCre^*R26*^N1c/+^ mice revealed increased CD22 expression upon treatment of the mice with Poly I:C (Fig. 5a). Expression of CD22 in Treg cells was abrogated upon Treg cell-specific deletion of *Rbpj*, the gene encoding the Notch canonical pathway transcriptional co-factor RPBJ (**Extended Data** Fig. 4a). Analysis of peripheral blood Treg cells of MIS-C subjects revealed increased expression of CD22 that strongly correlated with Notch1 expression on these cells (Fig. 5b). In contrast, CD22 was minimally expressed on Treg cells of control subjects or those of patients with acute COVID-19. CD22 was minimally expressed on CD4^+^ Tconv cells of control, acute COVID-19 and MIS-C subjects, and it did not correlate with Notch1 expression on these cells, indicating that it uniquely identified Treg cells with active Notch1 signaling (Fig. 5b,c).

To determine the role of CD22 expression on Treg cells in the multi organ inflammatory disease triggered by Poly I:C treatment of *Foxp3*^EGFPCre^*R26*^N1c/+^ mice, we examined the impact of therapy with a blocking anti-CD22 mAb on disease outcome in these mice. Anti-CD22 mAb treatment prevented the weight loss and the multi-organ inflammation induced by Poly I:C treatment. It downregulated the activation of splenic CD44^+^CD62L^−^ Tconv cells and the expression by Treg and Tconv cells of IFNγ (Fig. 5d–h). Anti-CD22 mAb treatment also downregulated the expression by splenic Treg cells of α4β7 (Fig. 5j). In contrast, B cell depletion with an anti-B cell specific anti-CD20 mAb failed on its own to protect against disease or to abrogate protection by anti-CD22 mAb treatment (**Extended Data** Fig. 4b-c). Analysis of gut lamina propria lymphocytes (LPL) revealed increased infiltration with activated (CD44^+^CD62L^−^) Tconv and Treg cells with increased expression of IFNγ that was similarly downregulated upon treatment with the anti-CD22 mAb (**Extended Data** Fig. 4d-e). These results indicated that Notch1 canonical pathway-induced CD22 expression on Treg cells plays a crucial pro-inflammatory role in Poly I:C-treated *Foxp3*^EGFPCre^*R26*^N1c/+^ mice.

**Figure 6:**
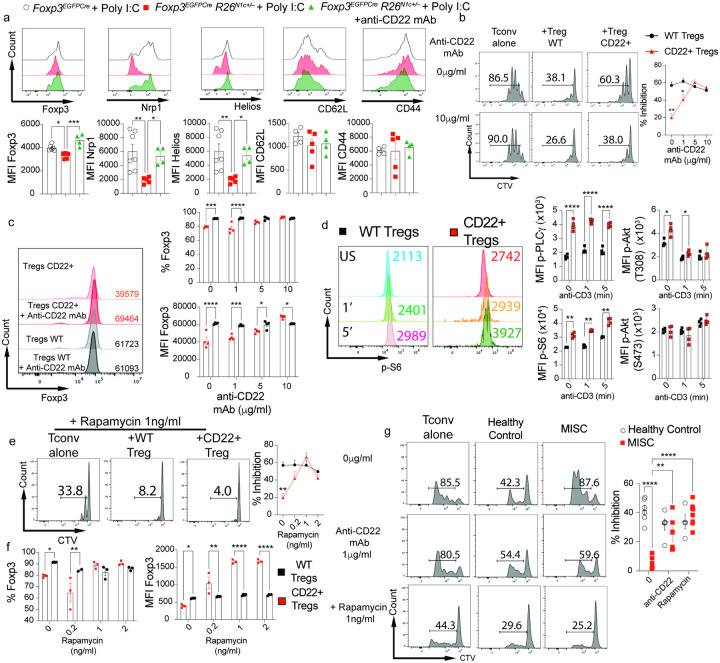
CD22 destabilizes Treg cells by an mTOR-dependent mechanism. **a**. Flow cytometric analysis and MFI of colonic Treg cell markers of Poly I:C-treated *Foxp3*^EGFPCre^ and *Foxp3*^EGFPCre^*R26*^N1c/+^ mice co-treated with isotype control mAb or anti-CD22 mAb, as indicated. **b**. *In vitro* suppression of Tconv cell proliferation by *Foxp3*^EGFPCre^ and CD22^+^ *Foxp3*^EGFPCre^*R26*^N1c/+^Treg cells in the presence of increasing concentrations of anti-CD22 mAb. c. Frequencies of Foxp3^+^ Treg cells and Foxp3 MFI in *in vitro* Treg cell cultures. **d**. Flow cytometric analysis and MFI of pPLC-g, p-T308-AKT, pS6 and p-S473AKT expression induced by anti-CD3 mAb treatment of *Foxp3*^EGFPCre^ and CD22^+^ *Foxp3*^EGFPCre^*R26*^N1c/+^Treg cells. **e**. *In vitro* suppression of Tconv cell proliferation by *Foxp3*^EGFPCre^ and CD22^+^ *Foxp3*^EGFPCre^*R26*^N1c/+^Treg cells in the presence of increasing concentrations of Rapamycin. **f**. *In vitro* suppression of human Tconv cell proliferation by Treg cells isolated from healthy controls or MIS-C subjects either in the absence of presence of anti-CD22 mAb or rapamycin. Each symbol represents one mouse (a, d), or one cell culture each derived from a different mouse/human subject (b,c, e-g). Numbers in flow plots indicate percentages or MFI. Error bars indicate SEM. Statistical tests: One-way ANOVA with Dunnett’s post hoc analysis (a); Two-way ANOVA with Sidak’s post hoc analysis (b-g); *P<0.05, **P<0.01, ***P<0.001, ****P<0.0001.

To determine the mechanisms by which CD22 subverted Treg cell function, we analyzed the steady state transcriptome of CD22^+^ Treg cells of *Foxp3*^EGFPCre^*R26*^N1c/+^ mice compared to control *Foxp3*^EGFPCre^ Treg cells or CD22^−^ Treg cells of *Foxp3*^EGFPCre^*R26*^N1c/+^. KEGG/GO pathway analysis showed increased expression of genes involved in the regulation of the immune response, T cell migration and Notch signaling (Fig. 5a–c). Furthermore, we analyzed the phenotypes of CD22^+^ colonic and splenic of Treg cells *Foxp3*^EGFPCre^*R26*^N1c/+^ mice isolated at steady state and following Poly I:C-treatment with those of Treg cells of similarly treated *Foxp3*^EGFPCre^ mice. The CD22^+^ Treg cells exhibited decreased expression of Helios and NRP-1 both at steady state and after Poly I:C treatment in the face of similar expression of markers of T cell activation including CD44, suggesting their decreased stability. In agreement with this conclusion, Foxp3 expression also decreased in CD22^+^ Treg cells following Poly I:C treatment. Treatment with anti-CD22 mAb reversed those defects (Fig. 6a **and Extended DATA** Fig. 5). In vitro Treg cell suppression assay revealed profoundly defective suppressive function of CD22^+^ Treg cells compared to control, which was also corrected upon treatment of the cells with the anti-CD22 mAb (Fig. 6b). Anti-CD22 mAb treatment corrected the decreased cell frequencies and MFI of Foxp3 found at the end of the tissue culture period, indicative of reversal of CD22^+^ Treg cell instability (Fig. 6c).

CD22 has been described to regulate B cell receptor signaling by forming a molecular scaffold that enables coordinated docking of different downstream signaling pathways^53^. Analysis of CD22^+^ Treg cells revealed enhanced T cell receptor signaling compared to control Treg cells, with increased phosphorylation of the, extracellular signal-regulated kinases (ERK)^54^ and the phospholipase C gamma 1 (pPLCγ1) (Fig. 6d **and Extended** Fig. 5). Downstream of the PI3-kinase pathway, phosphorylation of the kinase AKT at residue T308, a target of upstream phosphoinositide-dependent kinases, and the mammalian target of rapamycin complex 1 (mTORC1) substrate S6 kinase were also increased (Fig. 6d)^55^. In contrast, AKT phosphorylation at residue S473, a target of mTORC2, was unchanged^56^. Treatment with the mTOR inhibitor Rapamycin reversed the regulatory defect in CD22^+^ Treg cells and the associated loss of Foxp3 expression (Fig. 6e–f). Finally, In vitro Treg cell suppression assay from MIS-C patients also revealed profoundly defective suppressive function compared to Healthy control, which was also corrected upon treatment of the cells with the anti-CD22 mAb or Rapamycin (Fig. 6g). These results indicated that CD22 positively enhanced T cell receptor signaling in Treg cells leading to Treg cell destabilization and loss of regulatory function by an mTORC1-dependent mechanism.

## Discussion

In this study, we demonstrate that the evolution of MIS-C entails the mobilization of a Treg cell-specific pathway involving Notch1-CD22 signaling that promotes immune dysregulation and which can be demonstrated both in human subjects and in proxy mouse models. Patients with MIS-C but not children or adults with acute COVID-19 demonstrated increased Notch1 expression on circulating CD4^+^ Treg and Tconv cells, which declined precipitously following anti-inflammatory therapy. The pathogenic function of this pathway was confirmed by the identification of dominant negative mutations in PTB domains of *NUMB* and *NUMBL* in MIS-C subjects that resulted in increased Notch1 expression. It was also supported by the demonstration of a role for Notch pathway-related mutations in MIS-C using Monte Carlo simulation and Fischer Test. Uniquely, MIS-C subjects exhibited increased CD22 expression on Treg but not Tconv cells which could be demonstrated in mice to involve Notch1 signaling via the RBPJ-k canonical pathway. CD22 blockade was sufficient to inhibit the immune dysregulation triggered by Notch1 signaling in Treg cell in the Poly I:C proxy viral infection model, highlighting the critical role of this molecule in MIS-C disease pathogenesis.

Our previous studies have identified NOTCH4 to be specifically upregulated on circulating Treg cells in adult subjects with COVID-19; their origin could be traced in mouse models of viral infection to the lung^30^. NOTCH4 was similarly upregulated on circulating Treg cells of pediatric subjects with acute COVID-19, while both NOTCH4 and NOTCH1 were upregulated on those of MIS-C subjects. However, expression of NOTCH1 and NOTCH4 on the Treg cells of the latter group was mutually exclusive, suggesting the respective Treg cell populations were ontogenically distinct. These findings suggest that NOTCH4 and NOTCH1 regulate distinct checkpoints in the evolution of immune dysregulation following SARS-CoV-2 and other viral infections. Thus, NOTCH4 appears critical for licensing lung inflammation following SARS-CoV2, influenza and related viral infections^30^. In contrast, NOTCH1 may favor the spread of the initial lung inflammation systemically. Such a division of labor is supported by previous studies showing that the induction Notch1 on Treg cells plays a pathogenic role in mouse models of graft versus host disease and cardiac allograft rejection^34, 35^.

Mouse studies revealed that a critical step by which Notch1 signaling in Treg cells promotes systemic inflammation involves its induction of CD22, an inhibitory receptor previously associated with B cells, where it functions as a regulator of B cell receptor signaling, more recently, CD22 has been described to direct the homing of B cells to the gut lymphoid and mucosal tissues by virtue of its upregulation of the gut homing integrin α4β7. Consistent with these findings, treatment of mice whose Treg cells express a gain of function Notch1 mutant with an anti-CD22 blocking antibody rescued their gut and systemic inflammation following treatment with Poly I: C. CD22 impaired the *in vitro* Treg cell suppressive function, an effect that was reversed by treatment with the anti-CD22 blocking antibody. Notwithstanding its function as an inhibitory receptor, Treg cells expressing CD22 demonstrated T cell receptor signaling and heightened proliferation.

MIS-C is a rare complication of SARS-CoV-2 infection^57^, suggesting a genetic predisposition to this disorder. In that regard, mutations in a number of immune regulatory genes have been described in MIS-C, including *SOCS1*, *XIAP* and *CYBB*^26^. We found variants in a number of Notch pathway genes in patients with MIS-C. Importantly, dominant negative loss of function mutations in *NUMB* and *NUMBL* found in MIS-C subjects resulted in increased NOTCH1 expression and NOTCH1 signaling, consistent with the pathogenic function of mutations in this pathway in disease pathogenesis.

Collectively, these results allow for the construction of a model that traces the evolution of MIS-C. An initial infection with the SARS-CoV2 virus results in the expansion of NOTCH4 and NOTCH1 Treg cells, the latter favored by rare genetic variants found in the susceptible host. A subset of NOTCH1^+^ Treg cells upregulates CD22, which severely impairing their regulatory function and driving their homing to the gut where they promote inflammation possibly in a virus and microbiota-dependent manner. This cascading immune dysregulatory process may be amplified by heightened responses among some children to pathogen- and damage-associated signals^21^, which aggravates the systemic spread of inflammation and the broad disruption of tissue Treg cell function. This process is reversible by anti-inflammatory therapy that targets cytokines involved in Notch1 induction. Our results also suggest that blockade of CD22 may provide an alternative therapy in those patients who prove resistant to standard of care anti-inflammatory therapy.
